# Stability of the *w*Mel *Wolbachia* Infection following Invasion into *Aedes aegypti* Populations

**DOI:** 10.1371/journal.pntd.0003115

**Published:** 2014-09-11

**Authors:** Ary A. Hoffmann, Inaki Iturbe-Ormaetxe, Ashley G. Callahan, Ben L. Phillips, Katrina Billington, Jason K. Axford, Brian Montgomery, Andrew P. Turley, Scott L. O'Neill

**Affiliations:** 1 Bio21 Institute, Department of Genetics, The University of Melbourne, Parkville, Victoria, Australia; 2 School of Biological Sciences, Monash University, Clayton, Victoria, Australia; 3 Department of Zoology, The University of Melbourne, Parkville, Victoria, Australia; The Pennsylvania State University, United States of America

## Abstract

The *w*Mel infection of *Drosophila melanogaster* was successfully transferred into *Aedes aegypti* mosquitoes where it has the potential to suppress dengue and other arboviruses. The infection was subsequently spread into two natural populations at Yorkeys Knob and Gordonvale near Cairns, Queensland in 2011. Here we report on the stability of the infection following introduction and we characterize factors influencing the ongoing dynamics of the infection in these two populations. While the *Wolbachia* infection always remained high and near fixation in both locations, there was a persistent low frequency of uninfected mosquitoes. These uninfected mosquitoes showed weak spatial structure at both release sites although there was some clustering around two areas in Gordonvale. Infected females from both locations showed perfect maternal transmission consistent with patterns previously established pre-release in laboratory tests. After >2 years under field conditions, the infection continued to show complete cytoplasmic incompatibility across multiple gonotrophic cycles but persistent deleterious fitness effects, suggesting that host effects were stable over time. These results point to the stability of *Wolbachia* infections and their impact on hosts following local invasion, and also highlight the continued persistence of uninfected individuals at a low frequency most likely due to immigration.

## Author Summary

The *w*Mel infection is a *Wolbachia* infection introduced into *Aedes aegypti* mosquito populations; *w*Mel can provide potential suppression of dengue if the infection persists in populations. We show that the infection has persisted at a high frequency since its initial introduction into two natural populations in North Queensland, Australia. The infection, which is inherited through females, appears perfectly transmitted to offspring in the field. It has continued to produce a high level of incompatibility when infected males are mated with uninfected females; these females are likely to enter populations at a low frequency as migrants. These features ensure that the infection will stay at a high frequency in populations, but costs prevent its rapid spread.

## Introduction

Insect populations can evolve to respond rapidly to infectious agents including endosymbionts. Examples include the evolution of host suppressors that alter sex ratio of endosymbionts that cause male killing in *Hypolimnas bolina* [1] and changes in the lifespan effects induced by *w*MelPop *Wolbachia* infection in *Drosophila melanogaster* attenuated through selection [2]. Yet in other cases the effects of endosymbionts on host characteristics seem stable. For instance male-killing *Wolbachia* in *Drosophila innubila* have been present for at least 15000 years and there has been no selection for resistance [3].

In addition to evolution in the host genome, changes can also occur among *Wolbachia* strains. These changes have been studied in the *w*Ri *Wolbachia* infection of *Drosophila simulans* first detected in the 1980s [4] and subsequently spread throughout the world [5]. During this process, there has been attenuation of deleterious effects of the *Wolbachia* on fecundity [6]. However there has been little change in cytoplasmic incompatibility caused by the infection or infection frequency in natural infected populations where a combination of maternal transmission leakage and cytoplasmic incompatibility dictates the expected frequency of the infection [7]. Moreover, genomic data point to little change in the *w*MelPop infection across 4 years after transfer into *A. aegypti* mosquitoes [8].

The stability of *Wolbachia* infections is critical if such infections are to be successfully used in insect host and disease control. *Wolbachia* infections have been proposed as a novel intervention for the long term to suppress dengue and other arboviruses in endemic areas [9], [10]. This strategy depends on *Wolbachia* infections being maintained stably at high levels within natural populations as well as continuing to exhibit virus interference without producing changes in virulence. Although the evolution of virulence is unpredictable, comparative data suggest that virulence is unlikely to increase [11]. However *Wolbachia* might still not stably persist in populations if there are changes in maternal transmission, cytoplasmic incompatibility and/or fitness effects associated with *Wolbachia* infections.

Recently the *w*Mel *Wolbachia* infection originally described from *Drosophila melanogaster* and transferred into *A. aegypti* mosquitoes was successfully introduced into two areas near Cairns [12]. This infection suppresses the ability of mosquitoes to transmit dengue [13] and other arboviruses [14], [15] and is expected to persist without further introductions because it causes cytoplasmic incompatibility (CI), the process whereby eggs of uninfected mosquitoes die when uninfected females mate with infected males [13]. The successful invasion points the way to the use of *Wolbachia* to control dengue in disease endemic areas.

Because of the possibility that changes might interfere with the invasion potential of *Wolbachia* and persistence of the infection, it is important to instigate long term monitoring regimes to detect change. Recent research [16] has indicated that dengue interference has not been affected in the period following introduction of the *Wolbachia*. Here we test for the stability of the infection and changes in host fitness effects in the recently invaded areas after 3 years. We also provide the first estimate of maternal transmission of the infection based on field reared material based on the notion that at least in *Drosophila* patterns in field reared hosts can differ substantially to those obtained with laboratory reared material [17].

## Methods

### Field maternal transmission data

Adult *A. aegypti* mosquitoes were collected from Gordonvale (GV) and Yorkeys Knob (YK), Queensland in January and April/May, 2013. The mosquitoes were collected from BG-Sentinel traps set in residential locations within each suburb. The live female mosquitoes were taken back to the laboratory and then placed into individual 200 mL containers containing 50 mL of water and covered with mesh. Wet sandpaper was placed around the sides of the container to provide an oviposition substrate. Eggs were collected daily and left to embryonate for at least two days. Once egg laying was complete, each female was stored in ethanol and frozen at −20 °C.

Eggs from each individual female were hatched separately in 500 ml of reverse osmosis water, yeast and TetraMin Tropical Fish food. Mosquitoes were reared in a controlled temperature laboratory at 26 °C with a 12∶12 L∶D cycle and 55–85% humidity. The larvae were reared until they reached 3rd-4th instar, then stored in ethanol and frozen at −20 °C.

All adult and F1 larvae were tested for *Wolbachia* infection, using the methods previously described in Lee et al. [18]. Adults and larvae were extracted using a 5% Chelex-based method. This involved extracting the whole organism in 1.7 mL tubes using 3 µL of Proteinase K, 250 µL of Chelex solution with two 3 mm glass beads for adults and 150 µL of Chelex solution with four 2 mm glass beads for larvae. This was followed by grinding in a mixer mill. The tubes were then incubated for one hour at 65 °C and then for 10 minutes at 90 °C.

Polymerase chain reactions (PCR) were carried out using the Roche LightCycler 480 system (384-well format) using the RT/HRM (real-time PCR/high-resolution melt) assay [18]. This assay involves using three primers sets which include *Aedes* universal primers (*mRpS6_*F/*mRpS6_*R), *A. aegypti* specific primers (*aRpS6_*F/*aRpS6_*R) and *Wolbachia* specific primers (*w1*_F/*w1*_R). The crossing point (C*p*) and melting point (T*m*) of the PCR products from each primer are used to diagnose infection with *Wolbachia*. A sample was scored as *Wolbachia* positive when there is robust amplification of all three primer sets *mRpS6*, *aRpS6* and *w1*, while an *A. aegypti* sample that is W*olbachia* negative would amplify only *mRpS6*, *aRpS6.* Each RT/HRM assay was run using three positive *Wolbachia* controls and three negative *Wolbachia* controls.

In total, there were 32 females (all from GV) producing a total of 303 F1 offspring in the January collection and 47 females (45 from GV, 2 from YK) producing a total of 498 F1 offspring in the April/May collection. Binomial confidence intervals for maternal transmission rates were computed following Zar [19].

### Fecundity and cytoplasmic incompatibility

To evaluate any changes in fecundity effects of the infection and the expression of cytoplasmic incompatibility, we established infected and uninfected populations from field-collected individuals in April-May 2013 from both YK and GV. Females were collected from BG-S traps by visiting the traps daily. The infected YK population was established from the offspring of 6 field collected females and was maintained in the laboratory at a size of 450 individuals before being used in experiments. The uninfected YK population was initiated from a single inseminated female that produced offspring. Unfortunately compatible uninfected females are rare in the field.

For GV the numbers of field females used to establish the colonies were 34 for the infected colony and 1 for the uninfected colony. We also established an infected strain on a diverse Cairns genetic background. This strain (*w*C20) was the original release strain backcrossed again for 3 generations to field uninfected males from various parts of Cairns.

To test fecundity of the GV and YK mosquitoes, the colonies were blood fed (en masse) and then females were isolated individually from the cage (only blood fed fully engorged females were collected). These females were three generations removed from the field. Females from all treatments were fed on the same human volunteer to remove host effects (as approved by the University of Melbourne Human Ethics Committee (approval 0723847)). Engorged females were placed into 70 ml specimen cups containing 30 ml of water and sandpaper strips lining the inside of the container. Ten females were set up for each infected and uninfected colony from GV and YK, and 5 females from the *w*C20 colony were also included. Three days later eggs were collected and for 3 days thereafter. Eggs were collated across days. Four females did not lay or only laid a few eggs and these were excluded.

The fecundity data were analyzed with a two-way ANOVA run in IBM SPSS Statistics 22. Only the YK and GV data were included, with population and infection treated as fixed factors. We also analyzed the number of larvae emerging from the effects in the same way. Data were normally distributed by Kolmogorov-Smirnov tests, but we also analyzed the egg and larval numbers after log transformation and this transformation did not affect the outcome of the analyses.

### CI 2010 versus post-release

For cytoplasmic incompatibility, we set up 1.5 l containers each with 7 females and 7 males which were collected as virgins by separating them individually at the pupal stage. Sex was determined at the adult stage by exposing mosquitoes to 4 °C for 1 min to immobilize them. Eight containers (each with 7 females and 7 males) were set up per cross, and 9 crosses were undertaken (Table 1) based on different combinations of the infected and uninfected populations. The mosquitoes were first blood fed on the same human volunteer 9 days after eclosion and then 3 days later eggs were collected using sandpaper ovistrips which were replaced daily and collected over 4 days. This was followed by a second blood feeding with the same volunteer at 16 days with repeated oviposition and a third blood feed (again with the same volunteer) at 23 days. Eggs were conditioned after each laying period by drying eggs for 3 days before immersion into 500 ml of water with yeast added and ¼ tablet of TetraMin fish food. Hatch rate was scored after a week and containers were scored for larvae based on between 127 and 369 eggs per cycle.

**Table 1:** Crosses performed between infected and uninfected lines.

	Gonotrophic cycle		
	1	2	3
Incompatible			
*w*GV (♂) × GV (♀)	0 (284.25)	0 (268.12)	0 (302)
*w*GV (♂) × C20 (♀)	0 (321.75)	0 (369.25)	0 (221.62)
*w*C20 (♂) × GV (♀)	0 (259)	0 (267.5)	0 (222.5)
*w*YK (♂) × YK (♀)	0 (251.62)	0 (220.87)	0 (236.5)
*w*YK (♂) × C20 (♀)	0 (274)	0 (303.12)	0 (272.62)
*w*C20 (♂) × YK (♀)	0 (236.87)	0 (186.62)	0 (127.37)
*w*C20 (♂) × C20 (♀)	0 (302.12)	0 (322.75)	0 (270)
Compatible			
GV (♂) × *w*GV (♀)	0.77 (281.87)	0.88 (259.12)	0.73 (206.75)
YK (♂) × *w*YK (♀)	0.61 (162)	0.79 (262.12)	0.66 (166.25)

The “*w*” denotes infected lines. Compatible crosses are presented separately. Entries are hatch rates, with the average number of eggs per cross scored given in brackets.

### Field analysis of spatial distribution

BG traps were established throughout the main release areas and mosquitoes were retrieved weekly between 23 April 2012 and 25 October 2013. At YK, 44 trap locations were used in total across this period, but at any point in time mosquitoes were only retrieved from a maximum of 19 traps placed throughout the site (minimum 3 traps, mean 12). At GV, 45 trap locations were used, and mosquitoes retrieved from a maximum of 40 traps in the site (minimum 7, mean 19). Although some traps remained in the same location throughout the trapping period, several were moved when residents no longer wanted them on premises, or when they failed to catch adults over an extended period.

When traps were moved, they were usually moved to nearby locations, leading to around 40 unique localities at each site, which varied in their spacing and spatial independence. To account for the likely non-independence of trap locality, and to get a sense of the spatial scale at which independence arises between localities, we used a spatially-explicit point-level error structure (described below). Otherwise, our analysis consisted of a standard logistic regression in which we treat our observations (the number of *Wolbachia*-infected mosquitoes at location *i* and time *t, n_it_*) as arising from a binomial distribution: 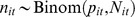
where *p_it_* is the probability of an individual being infected, and *N_it_* is the number of individuals sampled.

We then use a linear model to describe *p_it_* as, 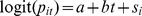
where *a* is the intercept (initial *Wolbachia* prevalence), *b* describes the change in prevalence over time, and *s_i_* is the spatially-explicit random effect.

Under this random effect, the covariation between sites scales with the distance between sites. To achieve this scaling, we chose a first-order covariance function, 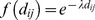, where *d_ij_* is the distance between site *i* and site *j*, and controls the rate of decay with distance. The resulting covariance matrix was then scaled by a coefficient of variation, *v*. Thus the vector of random effects is drawn from a multivariate normal distribution, 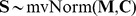



where **M** is a vector of zeros, and **C** is a variance-covariance matrix with elements (*c_ij_*) described by 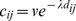



We fitted this model separately for each population using the JAGS Gibb's sampler [20], and used minimally-informative priors (see Table 2, Figs. S1, S2 in Text S1). We sampled the posterior distribution of our parameters using 30,000 samples from each of three chains following a burn-in of 30,000 samples. Convergence was assessed by examination of trace plots for each of the parameters. For simplicity, these analyses were run on data from both sexes combined because our initial logistic regressions on binomial data (depending on whether individuals were infected or uninfected) did not indicate any interactions between sex and time (P>0.3).

**Table 2:** Fitted spatially-explicit point-level models for each site.

Gordonvale				
Parameter	Prior	Posterior Quantile		
		2.50%	50%	97.50%
Initial prevalence, *a*	N(0, 1000)	3.65	4.20	4.75
Coefficient of time, *b*	N(0, 1000)	−0.0031	−0.0018	−0.0006
Variance due to sampling locality, *v*	1/Gamma(0.001, 0.001)	0.089	5.686	1416.015
Decay rate of across-locality covariance, *λ*	Gamma(0.001, 0.001)	0.0001	2.2621	1471.5936

Yorkey's Knob				
Parameter	Prior	Posterior Quantile		
		2.50%	50%	97.50%
Initial prevalence, *a*	N(0, 1000)	2.21	2.67	3.14
Coefficient of time, *b*	N(0, 1000)	−0.0011	0.0002	0.0015
Variance due to sampling locality, *v*	1/Gamma(0.001, 0.001)	0.023	2.186	2428.956
Decay rate of across-locality covariance, *λ*	Gamma(0.001, 0.001)	0.0074	8.8932	1151.1250

Minimally informative priors were used in all cases, and the central estimate as well as 95% credible interval (2.5–97.5%) is reported for the posterior of each parameter.

We also examined spatial patterns on data accumulated across time for individual traps. These analyses were run separately in the two time periods for GV when infections changed (see below). We used ArcGIS 9.3, R (“ape” and “vegan” libraries) and SAM v. 4.0 (Spatial Analysis in Macroecology) [21] in these analyses. To test for spatial patterns in infection frequencies across pooled data, we initially used the overall value of Moran's I computed in R, and then when there was significant spatial structure we computed Moran's I for different distance classes and used 1000 permutations run in SAM to assess the significance of spatial structure at different scales. The association between infection frequency in the traps and geographic distance was also compared through Mantel tests run in SAM. Spatial differences in infection frequencies between locations were visualized through mapping frequencies against trap location points in ArcGIS.

## Results

### Field maternal transmission data

For the January collection, there were 303 out of 303 positive detections. For the April/May collection, 498 out of 498 were positive detections. In all cases the negative controls indicated no *Wolbachia* and the positive controls all indicated *Wolbachia*. These results suggest perfect or near perfect maternal transmission in the field, with lower confidence intervals of >98% for the January collection and >99% for the April/May collection.

### Fitness – fecundity

**Figure 1:**
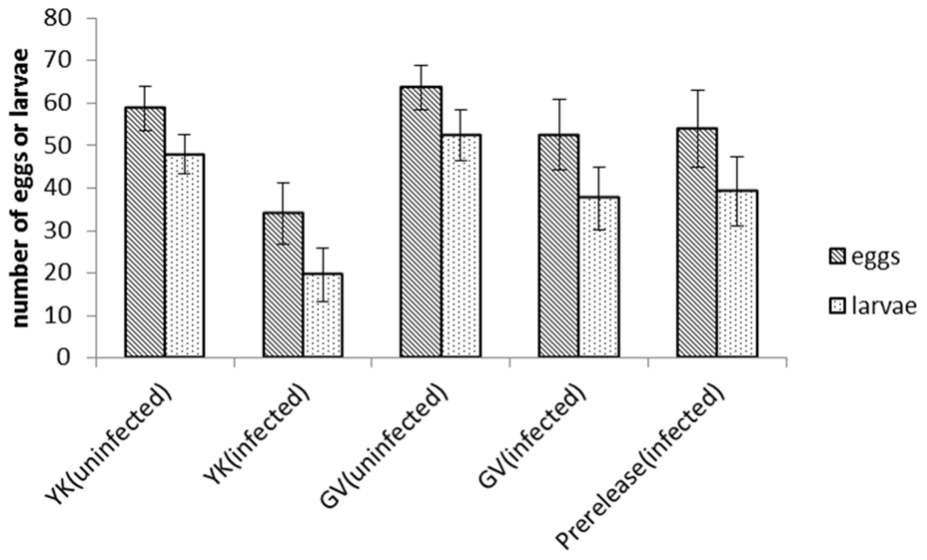
Egg and larval counts of females tested individually in containers. Error bars are standard errors.

The fecundity results (Fig. 1) indicate a reduction in egg numbers laid and larvae produced by the infected females compared to uninfected females from GV and YK. In the ANOVA involving only GV and YK, there was a significant effect of the infection (F_(1,31)_ = 7.32, P = 0.011) but no effect of population (F_(1,31)_ = 2.94, P = 0.096) or interaction between population or infection (F_(1,31)_ = 0.85, P = 0.364). There was a tendency for YK mosquitoes to have lower counts than GV mosquitoes but the results were quite variable. Mosquitoes from the infected line maintained in the laboratory showed similar egg counts to those from the infected GV population but these tended to be higher than for YK, which may reflect the fact that the YK lines were initiated with fewer field-collected mosquitoes. For larval counts, the ANOVA indicated a similar pattern with a significant effect of the infection (F_(1,31)_ = 12.51, P = 0.001) but not population (F_(1,31)_ = 3.22, P = 0.082) or an interaction (F_(1,31)_ = 1.11, P = 0.301). These results suggest that the negative effects of the *w*Mel infection on fecundity and larval production have persisted in time.

### Cytoplasmic incompatibility

For the compatible crosses, hatch rates were initially 76 and 61%, which then increased in the second gonotrophic cycle and then decreased into the third cycle (Table 1). For the incompatible crosses, no eggs were observed hatching for the first gonotrophic cycle. For each of cycle 2 and 3 there were 2 hatched eggs out of several thousand eggs scored, which did not develop beyond the first instar stage and were therefore scored as incompatible. These results highlight that incompatibility associated with infected males from the field and susceptibility to CI in uninfected females remained complete.

### Field analysis of spatial distribution

#### Yorkeys Knob

We had poor resolution on both the scale of spatial independence (controlled by *λ*), as well as the random effect of sampling locality (controlled by *v*; Table 2). This poor resolution is not unexpected given that the data are often at the very bounds of the probability scale (mean prevalence across all sites was 0.96), but make it impossible to be clear about the scale of spatial independence in these data. Nonetheless, our best estimate of lambda suggested a spatial covariance that declines to zero at distances around 500 m (Fig. S3 in Text S1) indicating relatively localized structure.

**Figure 2:**
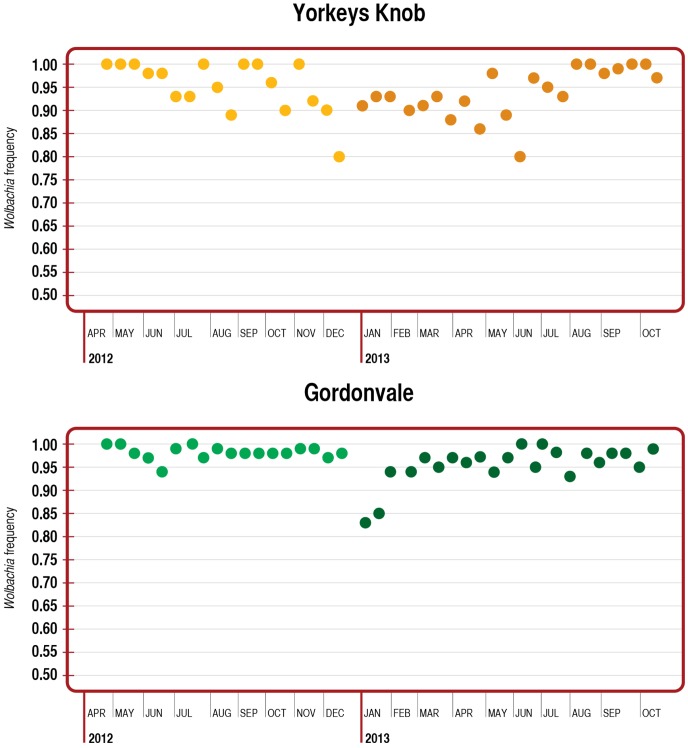
Changes in frequency of uninfected mosquitoes across time within Yorkeys Knob and Gordonvale release sites. Only frequency estimates from samples > 18 are given.

**Figure 3:**
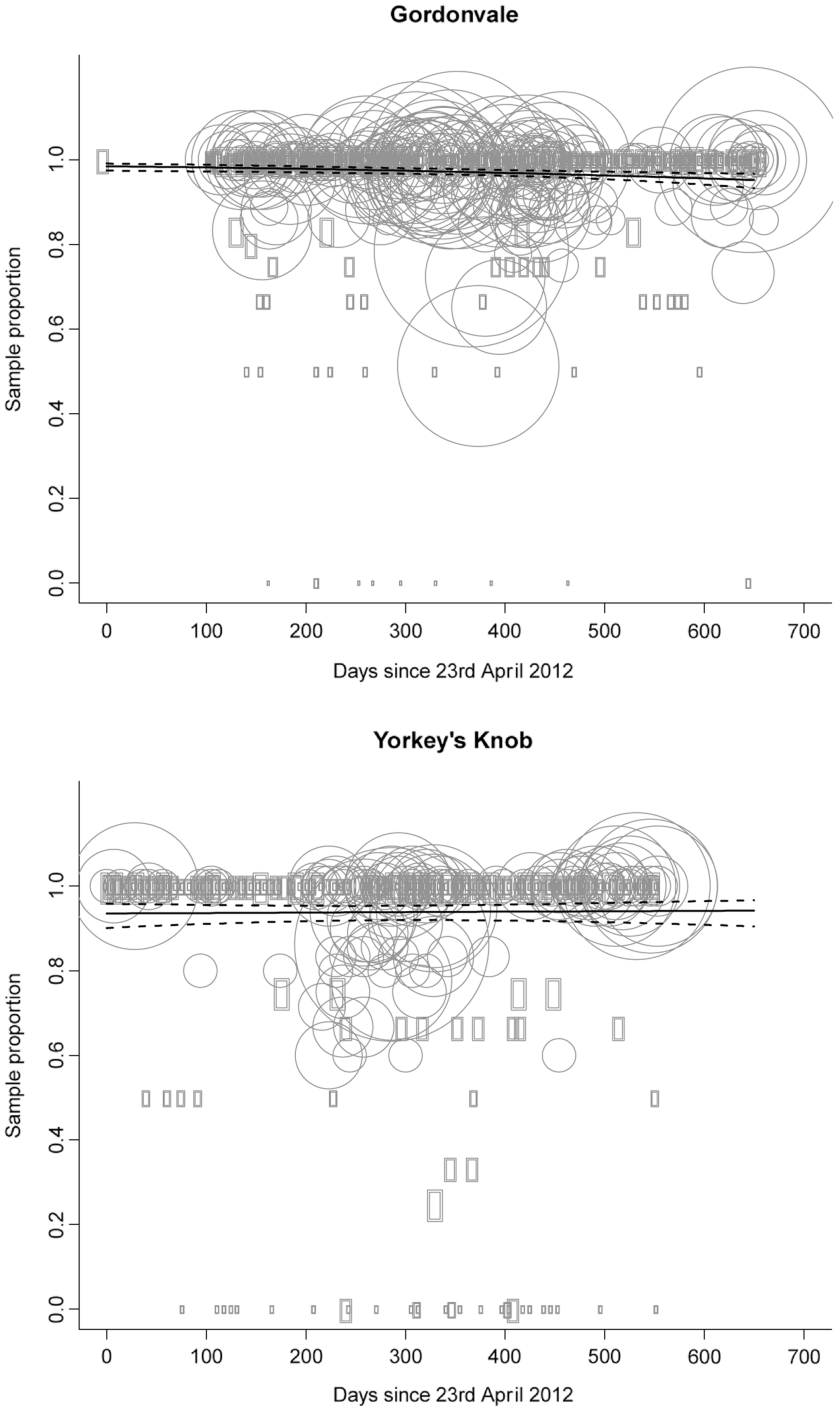
Sampled prevalence, and trends in prevalence across time within Yorkey's Knob and Gordonvale release sites. The circles represent the raw data – proportion of infected mosquitoes – and the size of each circle denotes relative sample sizes at each site (ranging from 1–47 mosquitoes per sample at Gordonvale, and 1–29 at Yorkey's Knob). The trend line (solid line) and its 95% credible bounds (dashed lines) are also shown.

Despite the poor resolution including the spatial terms means that we have controlled for any spatial non-independence between our sampling localities. When we do so, it is clear that prevalence is apparently static at YK (95% credible interval, −0.0011–0.0015: Table 2, Fig. 2, Fig. S2 in Text S1). To more clearly display these temporal patterns, the number of uninfected and infected mosquitoes caught in the traps was combined across 2 week periods to ensure a minimum sample size of 18 mosquitoes (median  =  44, maximum  =  116) for comparing infection frequencies when looking for trends (Fig. 3). The infection frequency decreased in mid-2012, but then increased again in late 2013 to near fixation. The lowest frequency was observed in mid-December 2012 when it fell to 80.3% (N = 71, binomial confidence intervals 70.6–88.8%). The long-term average infection frequency was 94.0% (confidence intervals 92.9–95.0% based on total data).

**Figure 4:**
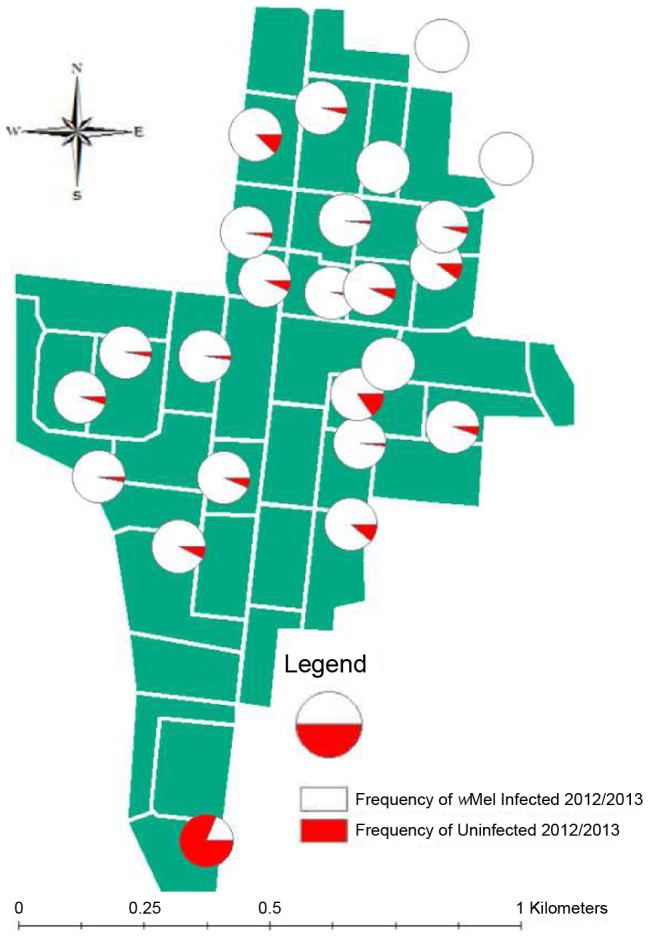
Distribution of infected (filled) and uninfected mosquitoes retrieved from BG-S traps after releases were terminated at Yorkeys Knob.

To characterize spatial patterns in infection frequency when pooled across time, we computed frequencies for all traps that had at least 12 individuals (median  =  36, maximum  =  405) and where traps had collected *A. aegypti* on at least 7 occasions (median  =  24, maximum  =  106). Across all traps, there was a significant difference in the number of infected versus uninfected mosquitoes collected (G = 122.7, df = 27, P<0.001 by Monte Carlo simulation). One trap at the road entry point of YK collected a large number of uninfected mosquitoes (13 out of 16, frequency  =  18.8%, confidence intervals 4.4–45.6 %) (Fig. 4). When this point was excluded, the infection frequency ranged from 84.6% to 100% in the traps.

In the spatial analysis using Moran's I, there was no evidence of spatial structure, regardless of whether the trap near the YK entry point was included or excluded from the analysis (P>0.5 in both cases). A Mantel test comparing infection frequencies across traps with geographic distance also failed to find a significant association when the entry point trap was excluded (r = −0.017, P = 0.915). In agreement with the individual time and location point analysis, uninfected individuals therefore appeared scattered throughout YK, perhaps with the exception of the trap at the entry point to YK.

#### Gordonvale

The infection frequency was higher at Gordonvale than Yorkeys Knob (Table 2; Fig. S1 in Text S1), but there was a downward trend in prevalence at GV (95% credible interval for the coefficient of time, −0.003–−0.0006). As in the case of YK, the best estimate of lambda suggest spatial covariance that declines to zero at distances around 500 m (Fig. S3 in Text S1).

We visualized these changes by combining mosquitoes across the 2-week intervals (Fig. 3), when the minimum number of mosquitoes per sample was 20 (median  =  139, maximum  =  248). The infection frequencies were mostly > 95% in 2012, but then dropped to 82.6% (N = 172, confidence intervals of 76.6–87.9%) in early 2013 and stayed low in the ensuing period before increasing again to values >90% after January. Considering only the 2012 data, the overall infection frequency was 98.2% (confidence intervals 97.7–98.6%) compared to the post-January frequency of 96.2% (confidence intervals 95.3–97.0%) suggesting a slight decrease in the infection rate in 2013.

**Figure 5:**
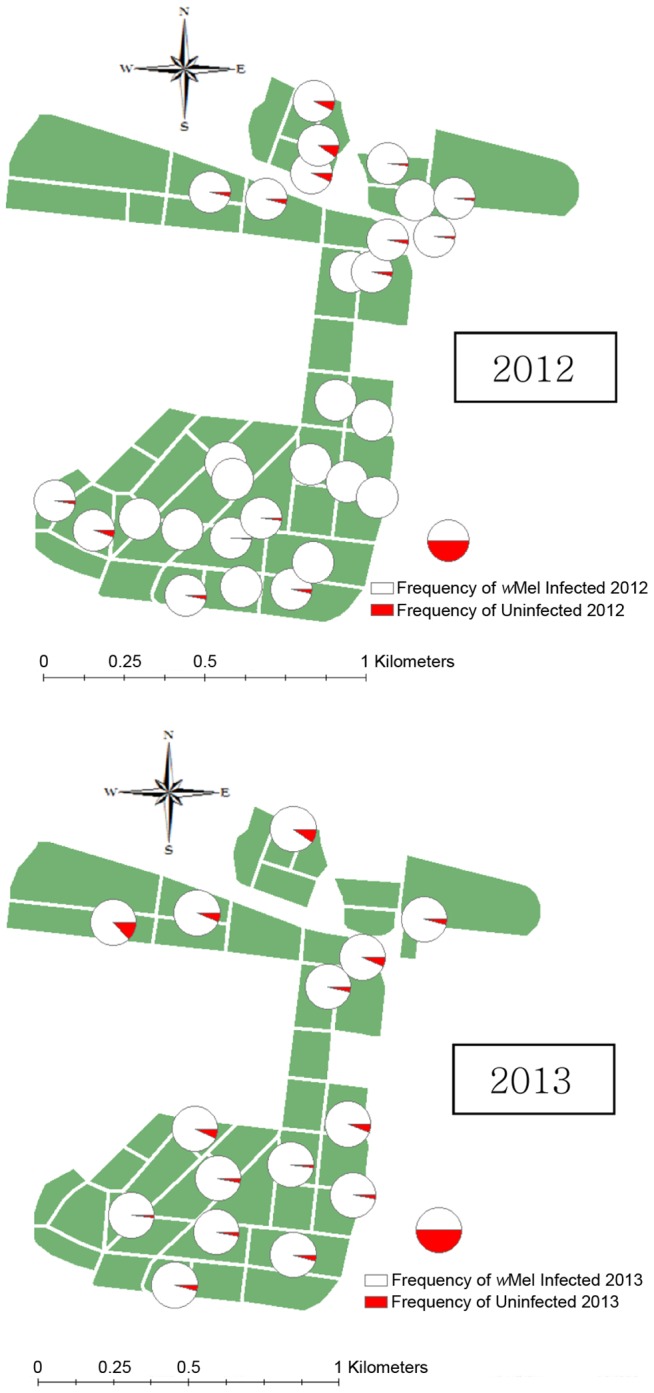
Distribution of infected (filled) and uninfected (unfilled) mosquitoes retrieved from BG-S traps after releases were terminated at Gordonvale across 2012 and 2013. Red areas indicate the frequency of the infected mosquitoes at each point.

For the Moran and Mantel tests, we computed frequencies for all traps that had at least 20 individuals across 7 occasions and analyzed data separately for the 2012 and post January 2013 periods given the changes in frequency mentioned above. For 2012, the median number of mosquitoes per trap was 78 (maximum 520) while for 2013 it was 96 (445). Individual trap frequencies ranged from 91.1% to 100% (Fig. 5).

There was significant spatial structure based on Moran's I for both 2012 (I = 0.196, P<0.001) and 2013 (I = 0.047, P = 0.025). In the Mantel tests, there was a significant association between the infection frequency and geographical distance in both 2012 (r = 0.255, P = 0.010) and 2013 (r = 0.359, P = 0.005). The analysis of Moran's I at individual distances suggested traps <300 m apart had a similar frequency of the infection. Areas with a relatively high frequency of uninfected individuals overall tended to occur in the northern part of GV (Fig. 5).

## Discussion

The long-term data collected from these areas 2–3 years after releases were initiated, indicate a stable pattern of *Wolbachia* invasion. Although uninfected mosquitoes persist in the areas, the original invasion which led to a rapid increase in infection frequency over a few weeks has been successfully maintained. The infection also has not spread outside the surrounding area at this time, most likely reflecting the relative isolation of these areas and the unstable equilibrium that is likely to exist to prevent an outward spread of *Wolbachia* [13]. During the initial invasion, a few *Wolbachia* were detected in areas adjacent to the release area but these detections were transient [12] and invasion is not expected to occur into an area of high mosquito density [22].

The infection frequency data from Yorkeys Knob and Gordonvale suggest that there are differences in the processes affecting uninfected mosquitoes in the two areas. In YK, the frequency of uninfecteds tended to be higher overall and without any detectable spatial structure or a high incidence of infected in particular traps. The spatial structure in GV was also very weak although Moran's I, when aggregated across time, suggests a higher frequency of uninfecteds in at least one area of the township. The uninfected mosquitoes may reflect movement into the area, or a loss of infection due to imperfect maternal transmission or perhaps curing of infected larvae during development. How likely are these processes and can they contribute to the different dynamics of uninfecteds seen in the release area?

Gordonvale is more isolated than Yorkeys Knob, being located 9 km from the nearest southern residential area of Cairns, whereas YK is only 1–3 km from the adjacent areas of Holloways Beach and Trinity Park. Therefore greater movement may contribute to the higher incidence of uninfecteds in Yorkeys Knob. Local active or passive movement of mosquitoes is evident from mark-release-recapture experiments conducted in the Cairns area [23], genetic data [24] and the movement of infected individuals outside the release areas [12]. Once uninfected females enter an area, they may contribute uninfected individuals to a population if they are already mated, otherwise they are likely to mate with infected males and express incompatibility leading to inviable eggs. The level of incompatibility seems complete in *A. aegypti* infected with *w*Mel [13] so only mated females from the surrounding area are expected to have much impact. On the assumption that uninfecteds in the BGS traps arise only from these females and their offspring, this suggests a migration rate of around 3% for GV and 6% for YK. With an estimated adult population of 7–8000 for GV and YK based on data collected during the *Wolbachia* releases in the wet season [25], these results may indicate an invasion of 200–500 female mosquitoes into the adult population from the surrounding area.

Do the spatial and temporal patterns of uninfecteds provide any suggestion of migration or other processes contributing to uninfecteds? We found a high incidence of uninfecteds in the trap at the main entrance point of YK, although this did not extend to other traps. Uninfected mosquitoes were found at this trap at a markedly higher frequency than at other traps and this might reflect local movement. However there was no evidence of a higher incidence of uninfecteds at another point in GV near the highway where this might have been expected. If larval curing had been contributing to the uninfected population due to localized environmental conditions, the incidence of uninfected mosquitoes would have been expected to show a spatial pattern as was the case at Gordonvale but not at Yorkeys Knob. However local spatial patterns might also arise from entry points of mosquitoes from surrounding areas, such as through passive movement in vehicles.

We found no evidence that maternal transmission was leaky under field conditions. This represents the first estimate of maternal transmission under field conditions which was previously shown to be perfect under laboratory conditions [13]. Thus the situation in mosquitoes contrasts to the *w*Ri infection in *D. simulans* where maternal transmission was perfect in the laboratory [26] but there was leaky transmission in the field [27]. The perfect maternal transmission and CI mean that the persistence and spread of the infection will only be related to the fitness effect of the infection. Previous estimates of the deleterious fitness effects suggest that these are small for *w*Mel and that fecundity/viability costs are around 24% as estimated from field cages although these can be difficult to detect because of feeder effects on fecundity [13]. Although a direct comparison is difficult, our data for GV are based on one feeder and suggest a deleterious effect of a similar magnitude.

Overall, our data point to similar deleterious fitness effects and CI associated with the infection since the release. These results suggest that the unstable point for *w*Mel invasion may not have shifted much over three years, although the changes in infection frequency at GV suggest that the infection may not have reached a stable equilibrium yet. There is also no evidence for a shift in dengue interference effects across this period [16]. These results suggest that the *w*Mel infection will remain high across time once an invasion has been successful, and with ongoing fitness costs there is also unlikely to be rapid invasion into surrounding areas.

## Supporting Information

Text S1 Fig. S1: Posterior estimate of the intercept (parameter *a*) for both sites. Fig. S2: Posterior estimate of the change in prevalence with time (parameter *b*) for both sites. Fig. S3: Pairwise distances within sites (top panel) and decay of covariance with distance (lower panel). Dotted lines show 95% bounds around the decay function and reveal poor resolution with regard to the spatial scale of covariation operating in this dataset.
(PDF)
