# Supplementary material for: Stability of the wMel Wolbachia Infection following Invasion into Aedes aegypti Populations
Source: PLoS Negl Trop Dis. 2014 Sep 11;8(9):e3115. doi: 10.1371/journal.pntd.0003115 (PMC4161343; doi:10.1371/journal.pntd.0003115)
Supplement: Text S1 — Fig. S1: Posterior estimate of the intercept (parameter a) for both sites. Fig. S2: Posterior estimate of the change in prevalence with time (parameter b) for both sites. Fig. S3: Pairwise distances within sites (top panel) and decay of covariance with distance (lower panel). Dotted lines show 95% bounds around the decay function and reveal poor resolution with regard to the spatial scale of covariation operating in this dataset. (PDF) [file pntd.0003115.s001.pdf]

Supplementary Information — Stability of the wMel Wolbachia infection following invasion into *Aedes aegypti* populations

Ary A. Hoffmann, Inaki Iturbe-Ormaetxe, Ashley G. Callahan,
Ben L. Phillips, Katrina Billington, Jason Axford,
Brian Montgomery, Andrew P. Turley, Scott L. O'Neill

1 Supplementary Result Figures

The following figures (S1 to S2) are the estimated posterior densities of the key parameters a and b, and demonstrate the difference in initial prevalence and change in prevalence over time.

By contrast, the between-site coefficient of variation v, and the scale of spatial dependence, λ, although poorly resolved, appear to be quite similar across sites (see Table 1 in main paper). To visualise the way that covariation declines with distance, we provide figure S3 showing how standardised covariation declines with distance. To produce this figure, we mixed the posteriors for this parameter across the sites to generate a median and 95% credible interval for the parameter λ.

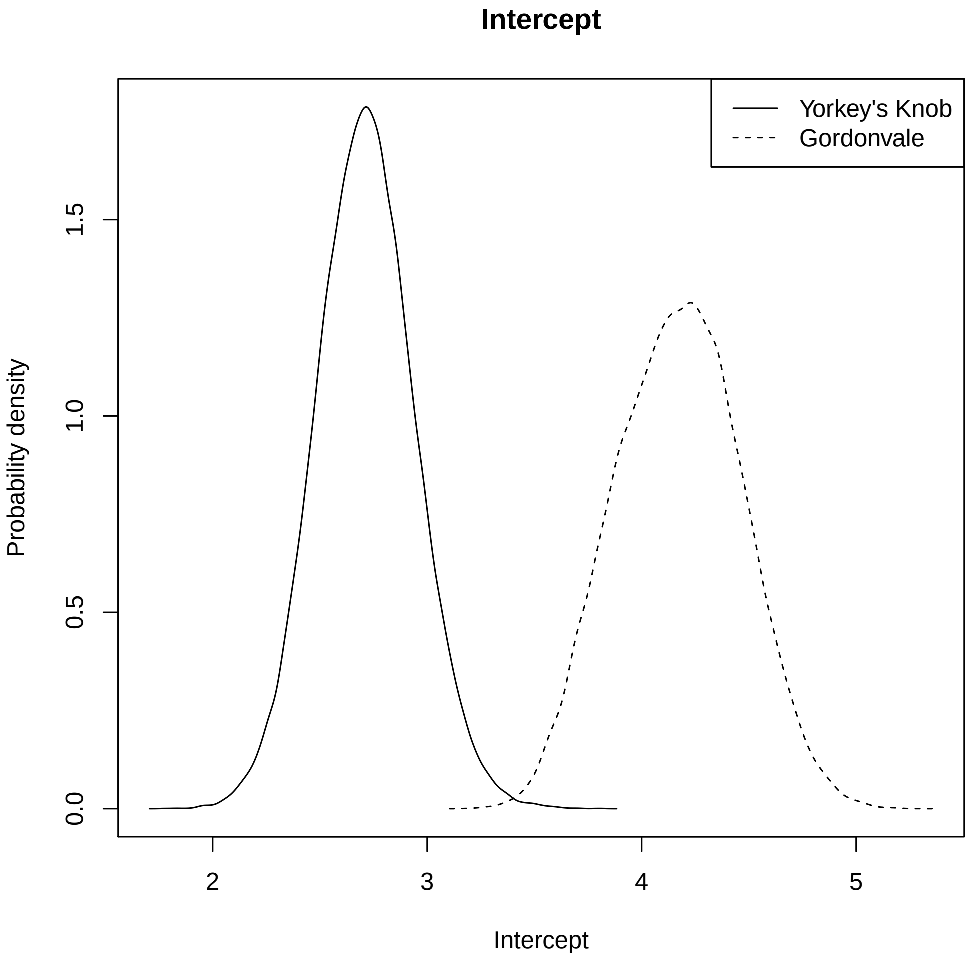

Figure S1: Posterior estimate of the intercept (parameter a) for both sites.

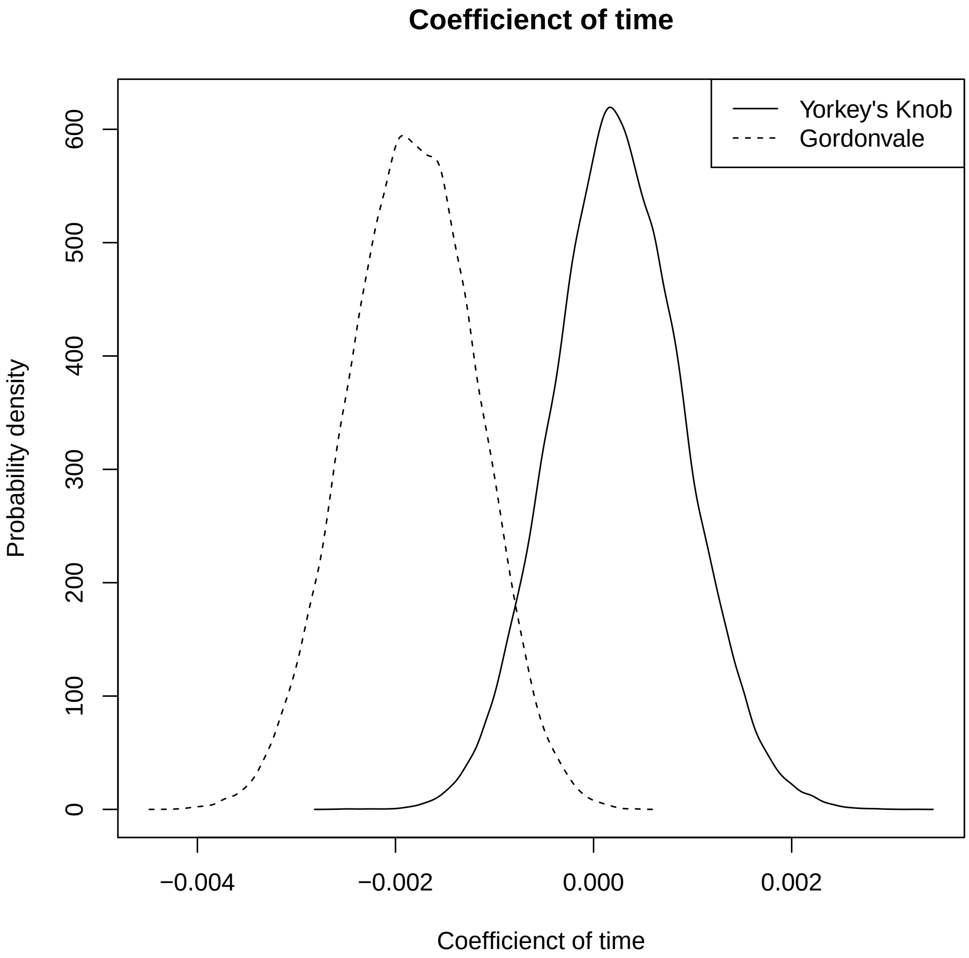

Figure S2: Posterior estimate of the change in prevalence with time (parameter b) for both sites.

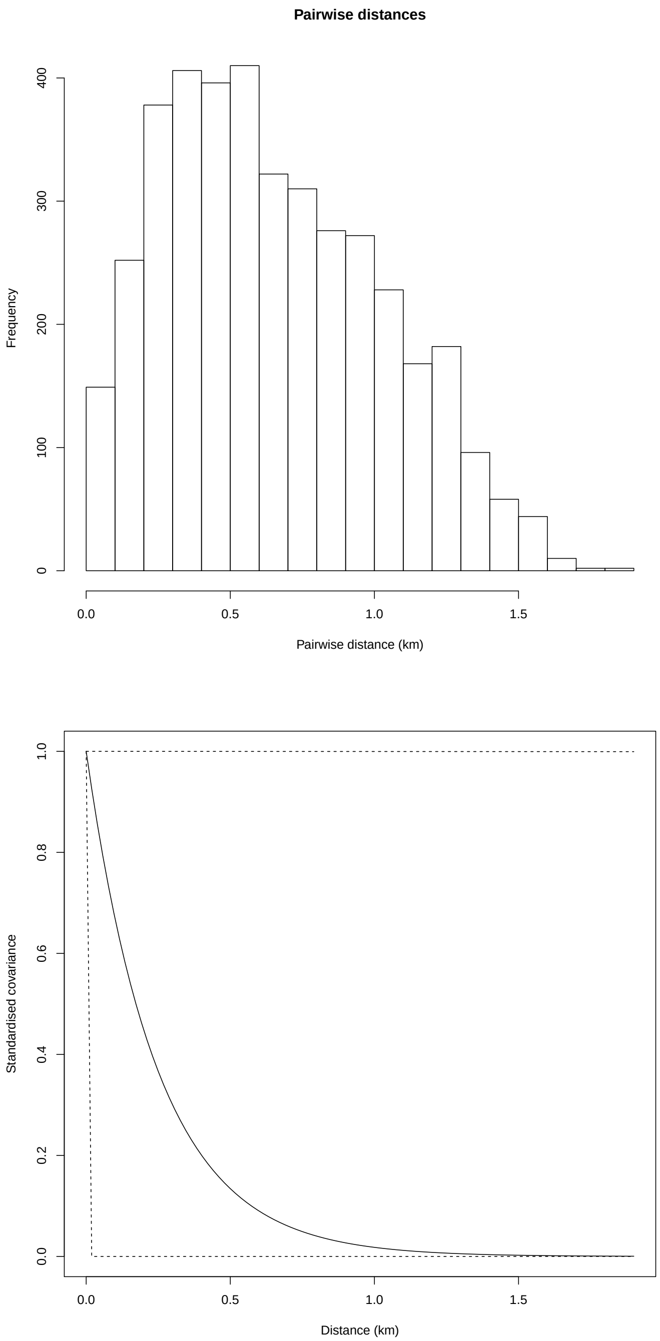

Figure S3: Pairwise distances within sites (top panel) and decay of covariance with distance (lower panel). Dotted lines show 95% bounds around the decay function and reveal poor resolution with regard to the spatial scale of covariation operating in this dataset.
